# Treating Depression in Older Adults: Challenges to Implementing the Recommendations of an Expert Panel

**Published:** 2007-12-15

**Authors:** Mark Snowden, Lesley Steinman, John Frederick

**Affiliations:** Department of Psychiatry and Behavioral Sciences, University of Washington School of Medicine; Department of Health Services, University of Washington School of Public Health and Community Medicine, Seattle, Washington; Department of Health Services, University of Washington School of Public Health and Community Medicine, Seattle, Washington

## Abstract

Depression is increasingly recognized as a significant public health problem among older adults. Because the condition is highly treatable and currently undertreated among community-based older adults, late-life depression is an appropriate focus for disease prevention programs. We report findings from a recent project to review the scientific literature for published reports about treatment for depression among community-dwelling older adults and to recommend the interventions with proven effectiveness. We also summarize the research findings related to each recommended intervention and describe the elements of each. To show the difficulties involved in translating research into practice, we describe real-world experiences in implementing these evidence-based interventions in various community settings. Because depression among older people is viewed more and more as a public health problem, we suggest that partnerships of providers, patients, and policy makers be forged to overcome challenges related to funding, training, and implementing treatments for this condition.

## Introduction

About 5% to 15% of community-dwelling older adults (i.e., adults aged 60 years or older) suffer from depression (1), which is associated with functional impairment (2-5), high health care costs (6,7), and possibly increased mortality rates through suicide and complications of cardiac disease (2-5,8,9). Recent data suggest that treatment can reduce not only depression but also the secondary symptoms such as pain and improve health-related quality of life (7-10). Whether treatment also reduces health care costs is unclear.

In light of the increasing burden of, and suboptimal treatment for, depression and the extensive scientific literature on treating and preventing depression (1,11), several major public health organizations designated depression as a major public health concern. For example, a key objective of *Healthy People 2010* is to reduce the proportion of adults with disabilities who report symptoms of depression and are less active because of those symptoms (12). In addition, the Task Force on Community Preventive Services endorsed depression as a topic for a systematic literature review to identify effective treatments (13). In this article, we report on a recent special interest project called Defining the Public Health Role in Depression in Older Adults (Depression in Older Adults project), which was supported by the Centers for Disease Control and Prevention (CDC) through the Prevention Research Centers' Healthy Aging Network (PRC–HAN).

## Methods

During the first stage of this project, an expert panel of 14 academics in public health or geriatrics (including two of the authors: MS, JF) systematically reviewed published, peer-reviewed studies to learn about successful interventions for depression among noninstitutionalized older adults. Panel members (who were recommended by CDC or PRC–HAN) reviewed all studies of interventions with the primary objective of reducing depression or interventions that had other primary objectives but evaluated depression as a secondary outcome (e.g., a study of participants in an aquatics class for elderly people with arthritis, after which researchers measured not only changes in participants' mobility but also changes in symptoms of depression).

The panel established the following eligibility criteria for studies to be included in our review: 1) the mean age of study subjects should be 60 years or older; 2) the number of subjects should be 25 or more; 3) subjects should not be institutionalized; 4) study criteria for determining whether participants were depressed were based either on a clinical diagnosis (e.g., major depression, dysthymia) or on a symptom-severity score from a standardized assessment instrument, and 5) the study report must clearly describe replicable interventions.

After the review was complete, the panel determined whether the study data were adequate to rate the intervention's effectiveness. When the data were adequate, panel members rated each intervention as effective, of mixed effectiveness, or ineffective. These determinations were based on the quality of the studies. Quality was based on, for example, dropout rates, adequacy of statistical analyses, and magnitude of study participants' response to the interventions. Full details about the criteria used to determine the adequacy of the data, effectiveness of the intervention, and quality of the studies are published elsewhere (14).

For the second stage of the Depression in Older Adults project, the panel was restructured: six of the original members left, and six community health care providers were added. This stage of the project is unique because the panel reviewing the literature and recommending interventions included not only researchers but practitioners familiar with the challenges of planning and implementing interventions. This second panel reviewed the list of interventions found through the literature review and recommended or strongly recommended certain of those interventions for treating late-life depression among community-dwelling older adults to healthy-aging experts and public health professionals. In selecting which interventions to recommend or strongly recommend, the panel considered not only their effectiveness but the feasibility and appropriateness of implementing them at the community level. The panel also suggested further research on promising interventions. The study methods and citations for reviewed studies are published elsewhere (14,15).

## Results

A total of 97 intervention studies met the panel's criteria for inclusion and were grouped into 24 intervention categories (Table). At the end of this two-stage project, the researcher-practitioner expert panel *strongly recommended* interventions based on the depression care management (DCM) model and *recommended* cognitive behavioral therapy (CBT) as treatment for depression in older adults. DCM was supported by eight randomized clinical trials (RCTs) with more than 3000 study subjects. These subjects experienced greater reductions in symptoms of depression, higher remission rates, and more improvement in health-related quality of life than did people in the control group who were given whatever care their physicians deemed appropriate. In addition, the DCM subjects often reported greater satisfaction with their care than did subjects given usual care. The review also found six RCT studies involving CBT. Typically researchers found that those given CBT treatment had significant improvement in their depression symptoms after less than 1 year. Further details on the reviewed studies and on interventions that are not recommended or that provided insufficient evidence are in the Table.

### Depression care management

The DCM model is a systematic team approach to treating depression in older adults, which is based on the model for treating chronic diseases (16). Common elements of DCM include diagnosing depression through a validated screening instrument and providing psychotherapy or antidepressants according to evidence-based guidelines. Treatment is reassessed periodically through a validated severity instrument to determine how well patients are responding and to adjust treatment if appropriate. A trained social worker, nurse, or other practitioner (sometimes called a "depression care manager" or "care manager") educates patients, tracks outcomes, facilitates psychotherapy, and monitors antidepressants prescribed by a primary care provider. The care manager works in consultation with a psychiatrist who supervises care but typically does not see the patients. The goal is to improve rates of adherence to treatment and to improve recognition of, and treatment for, patients not responsive to their initial treatment.

Managing depression in primary care clinics is effective: elderly people already visit these facilities regularly (17-19), and one study of depressed older adults found that DCM was delivered at a mean cost of $580 per patient (19), compared with total health care cost per patient of about $8000 (20). At-home interventions involve home visits by the depression care manager, who coordinates with other members of the collaborative care team outside the patient's home. One study of home-based management of depression found that costs averaged $630 per patient for an average of six visits (9).

### Cognitive behavioral therapy

CBT is psychotherapy that focuses on the clients' patterns of thoughts and behaviors that induce a depressed mood (21). The therapist teaches clients to recognize and modify these thoughts and behaviors in order to reduce symptoms of depression. CBT usually consists of weekly therapy sessions and daily exercises to help older adults apply CBT skills every day. Studies generally use trained therapists with master's degrees to deliver the intervention. The therapists are supervised by, and may consult with, professionals with a PhD or an MD.

## Real-World Experience

Several groups of experts recognize DCM and CBT as proven treatments for depression in many older adults (22,23), yet numerous obstacles prevent these interventions from being used by public health and healthy aging programs. Next we describe several efforts to implement the recommended evidence-based depression interventions in various communities.

### Depression care management

The Program to Encourage Active and Rewarding Lives for Seniors (PEARLS) is an example of a home-based program to manage depression (9). PEARLS began as a 5-year study of 138 subjects, during which research funds and administrative support were available for selecting and training interventionists, recruiting and funding a supervising psychiatrist, recruiting research subjects, collecting data, and assessing outcomes. After the study ended, community agencies began funding and supporting the program. The researchers continued their support through regular meetings with the agency staff and administrators to solve problems and to provide education and training.

As of April 2007, 35 community-dwelling older adults had completed treatment through a social service agency that serves homebound and frail older adults. These 35 were the first to complete treatment after the 5-year study ended. Their depression was diagnosed through the Patient Health Questionnaire (PHQ-9) (a nine-item, validated instrument for screening and diagnosing depression), and their initial average score (10.9) was similar to the initial average score of the participants in the 5-year study. A score of 10.9 indicates an intermediate level of depression (24). After treatment, the average PHQ-9 score of the 35 had decreased to 4.8 and 30 (87%) of the 35 were in remission. Unfortunately, the number of community-dwelling older adults treated (35) is small in comparison with the number of older adults enrolled in the social services program (2033) and the number of enrollees who have mild depression (at least 400). This situation shows that implementing the PEARLS intervention in a real-world setting (rather than a research setting) is difficult even when the obstacles of screening, funding, training, and staffing are overcome.

During a discussion among the researchers, administrators, and staff involved in PEARLS about the barriers to implementing the program more widely, several factors became evident. First, without research staff to recruit older adults with depression, the in-home case managers must identify older adults with depression and refer them to the PEARLS counselors. The case managers are responsible for many other aspects of a client's care, and most clients have needs in areas other than depression. Therefore, referring people with mild depression to PEARLS competes with many other case manager responsibilities. In addition, many clients, because of stigma or other reasons, do not see the need for treatment or are not interested in receiving treatment. Lastly, the research intervention protocol excluded people with moderate or high levels of cognitive impairment and people who did not speak English. The current PEARLS program has many such clients but does not have a blueprint for modifying and adapting the program to meet the needs of these diverse, real-world patients.

The Improving Mood–Promoting Access to Collaborative Treatment (IMPACT) study (http://impact-uw.org/) is an example of primary care, clinic-based DCM. The IMPACT study is the largest geriatric DCM trial conducted to date, involving 1801 older adults from 18 clinics in 5 states (19). The program trained nurses and psychologists to teach their patients problem-solving techniques, and the patients' primary care providers administered antidepressants as needed. Since the study's report was published in 2002, efforts to disseminate and implement the program have continued through a combination of in-person trainings, Web-based information and training modules, and grant-funded efforts to adapt the program to other settings or other populations (20). Although the number of people who received the intervention outside the research study is unclear, several states are collaborating with the study team to implement the program on a large scale.

IMPACT faces challenges similar to those that face PEARLS and other DCM programs. First, although primary care providers are comfortable using measurement-based care, primary care clinics do not usually screen for depression. Therefore, getting primary care providers to incorporate instruments such as PHQ-9 into routine care can be challenging. Second, although evidence clearly shows that nurses who are not health care specialists or nurse practitioners can function as care managers, most third-party insurance providers, including Medicare and Medicaid, do not reimburse expenses when registered nurses serve as care managers. Similarly, Medicare and Medicaid do not pay for a supervising psychiatrist. Finally, although the Internet has greatly reduced the challenges of training diverse audiences all across the country, it is unclear how much actual training is delivered through this mode of communication.

### Cognitive behavioral therapy

Cognitive behavior therapy is the oldest of the interventions recommended by the expert panel. Although some studies have been done on CBT (25), none were done in primary care settings or as part of community-based geriatric programs. However, since CBT is a single intervention technique, it does not face some of the challenges of multifaceted programs, which require several people to implement. CBT is usually taught during the intern and residency programs for psychiatrists, psychologists, and licensed clinical social workers. Because numerous self-help texts (26) are available detailing the theory and practice of CBT, many other mental health providers are familiar with its use. However, most of these practitioners work in specialty mental health settings removed physically from primary care or community-based programs that serve older adults. Therefore, linking the patient and the provider is a challenge because many older adults are reluctant to go to mental health specialists. In addition, the interventions that we determined were effective through the literature review were based on depression screening with quantitative instruments to guide and evaluate the therapy. This quantitative-based approach to delivering psychotherapy is not common in many mental health settings.

## Discussion

Two points from the review warrant further discussion. First, the panel did not find sufficient evidence from community-based studies to make any recommendations for therapies to deal with grief or prevent suicide. By excluding articles on studies that were based in academic settings, we may have amplified the problem of insufficient evidence. However, excluding these articles was consistent with the community-based focus of our review. Given the multiple losses experienced by older adults and the high suicide rate for older adults in the United States (27), more research is needed in these areas. Second, many of the reviewed interventions targeted primarily other conditions or outcomes (e.g., increases in physical therapy, training in certain skills) and measured depression levels only as a secondary outcome. These interventions did improve the targeted outcomes but did not alleviate depression. Therefore, although depression is a comorbid condition in many patients, it is an independent contributor to suffering and requires direct treatment.

Many real-world challenges to implementing the recommended depression interventions are also challenges for other areas of public health. These are acquiring adequate funds to set up and manage programs well, overcoming barriers to training staff in the intervention techniques, ensuring that people who need the service have access to it, ensuring staff fidelity to established protocols, and having adequate support to evaluate outcomes. Reducing the stigma attached to having mental health problems is one means of improving access to care, especially for older adults with depression. One advantage of the models for managing depression that we reviewed is that they can be made available in primary care clinics or in the home, places without any stigma attached to them.

In summary, several interventions are effective for treating depression in older adults and were deemed appropriate by an expert panel for community-based implementation. Many challenges remain, but overcoming these is an important public health priority. Partnerships among researchers, health care providers, and policy makers will be necessary to overcome the funding and training obstacles that block implementation of treatment programs for older adults. As shown by research studies (7-10) and stated by the President's New Freedom Commission on Mental Health, good emotional health is necessary for good physical health (28).

## Figures and Tables

**Table:** Intervention Categories, Data Adequacy, Effectiveness Rating, and Recommendations with Regard to Studies Reviewed for the Project: Defining the Public Health Role in Depression in Older Adults, 2004-2007

Intervention Category	Studies Reviewed (N)^a^	Adequate Data?	Effectiveness Rating	Recommendation
Depression care management (home)	8 (1119)	Yes	Effective	Strongly recommended
Depression care management (clinic)	2 (2399)	Yes	Effective	Strongly recommended
Group psychotherapy targeting depression	6 (292)	Yes	Ineffective	Insufficient evidence
Individual psychotherapies targeting depression: CBT	6 (432)	Yes	Effective	Recommended
Individual psychotherapies targeting depression: other therapies b (except CBT)	6 (490)	Yes	Mixed effectiveness	Insufficient evidence
Psychotherapy targeting mental health	5 (574)	Yes	Mixed effectiveness	Insufficient evidence
Psychotherapy for caregivers	2 (394)	Yes	Mixed effectiveness	Not applicable^c^
Education and skills training: targeting older adults	10 (2803)	Yes	Ineffective	Not recommended
Education and skills training: targeting caregivers	11 (2026)	Yes	Mixed effectiveness	Not recommended
Geriatric health evaluation and management (home)	7 (708)	Yes	Mixed effectiveness	Not recommended
Geriatric health evaluation and management (clinic)	4 (2157)	Yes	Ineffective	Not recommended
Exercise: primary target depression	1 (1828)	Yes	Not eligible^d^	Not eligible^d^
Exercise: other primary targets	9 (1796)	No	Mixed effectiveness	Not recommended
Bereavement: group therapy	2 (367)	Yes	Not eligible^d^	Not eligible^d^
Bereavement: hospice	1 (96)	No	Not eligible^d^	Not eligible^d^
Bereavement: individual treatment	1 (33)	No	Not eligible^d^	Not eligible^d^
Community-based suicide prevention	3 (18,641)	No	Not eligible^d^	Not eligible^d^
Suicide prevention: depression care management	1 (598)	No	Not eligible^d^	Not eligible^d^
Nutrition	1 (81)	No	Not eligible^d^	Not eligible^d^
Peer support	1 (291)	No	Not eligible^d^	Not eligible^d^
Adult day health	1 (44)	No	Not eligible^d^	Not eligible^d^
Incontinence	1 (30)	No	Not eligible^d^	Not eligible^d^
In-home respite for caregivers	1 (55)	No	Not eligible^d^	Not eligible^d^
Physical rehabilitation and occupational therapy	7 (822)	Yes	Ineffective	Not recommended

CBT indicates cognitive behavior therapy.

a The total number of participants in all studies reviewed in the category is given in parentheses.

b Other therapies include brief relational/insight therapy, brief psychodynamic therapy, self-management, reminiscence, bibliotherapy, and problem-solving.

c The second panel of reviewers moved studies originally categorized as "Psychotherapy for Caregivers" to the "Education and Skills Training Targeting Caregivers" category; therefore, no recommendation was made for interventions in the "Psychotherapy for Caregivers" category.

d Intervention categories for which data were inadequate were not eligible for an effectiveness rating or recommendation.
